# Resolving outbreak dynamics using approximate Bayesian computation for stochastic birth–death models

**DOI:** 10.12688/wellcomeopenres.15048.2

**Published:** 2019-08-30

**Authors:** Jarno Lintusaari, Paul Blomstedt, Brittany Rose, Tuomas Sivula, Michael U. Gutmann, Samuel Kaski, Jukka Corander

**Affiliations:** 1 Helsinki Institute for Information Technology (HIIT), Department of Computer Science, Aalto University, Espoo, Finland; 2 Department of Infectious Diseases Epidemiology and Modelling, Norwegian Institute of Public Health, Oslo, Norway; 3 Helsinki Institute for Information Technology (HIIT), Department of Mathematics and Statistics, University of Helsinki, Helsinki, Finland; 4 School of Informatics, The University of Edinburgh, Edinburgh, UK; 5 Department of Biostatistics, University of Oslo, Oslo, Norway; 6 Infection Genomics, The Wellcome Trust Sanger Institute, Hinxton, UK

**Keywords:** Approximate Bayesian computation, outbreak dynamics, stochastic birth–death process, tuberculosis.

## Abstract

Earlier research has suggested that approximate Bayesian computation (ABC) makes it possible to fit simulator-based intractable birth–death models to investigate communicable disease outbreak dynamics with accuracy comparable to that of exact Bayesian methods. However, recent findings have indicated that key parameters, such as the reproductive number *R*, may remain poorly identifiable with these models. Here we show that this identifiability issue can be resolved by taking into account disease-specific characteristics of the transmission process in closer detail. Using tuberculosis (TB) in the San Francisco Bay area as a case study, we consider a model that generates genotype data from a mixture of three stochastic processes, each with its own distinct dynamics and clear epidemiological interpretation.

We show that our model allows for accurate posterior inferences about outbreak dynamics from aggregated annual case data with genotype information. As a byproduct of the inference, the model provides an estimate of the infectious population size at the time the data were collected. The acquired estimate is approximately two orders of magnitude smaller than assumed in earlier related studies, and it is much better aligned with epidemiological knowledge about active TB prevalence. Similarly, the reproductive number *R* related to the primary underlying transmission process is estimated to be nearly three times larger than previous estimates, which has a substantial impact on the interpretation of the fitted outbreak model.

## 1. Introduction

Birth–death processes are flexible models used for numerous purposes, in particular for characterizing the spread of infections under the so-called Susceptible–Infectious–Removed (SIR) formulation of an epidemic process ^1^. Under circumstances where a disease outbreak occurs but where daily, weekly or even monthly incidence counts are not directly applicable or available, the estimation of key epidemiological parameters, such as the reproductive number *R*, has to be based on alternative sources of information. This can be the case when the disease demonstrates large variability between the times of infection and onset, such as with *Mycobacterium tuberculosis*, or in retrospective analyses where some information is no longer available. In such situations, aggregate measures of the clusteredness of cases (for instance, genotype fingerprints) can be used as alternative sources of information. In the case of tuberculosis, which generally mutates on a timescale much longer than that of a single outbreak, it is reasonable to assume that new cases arising from transmission during that outbreak will all belong to a single cluster. Likelihood-based inference could provide an alternative to standard outbreak investigations relying solely on incident count data, but it is often considerably more challenging.

As a solution to such a setting, Tanaka *et al* . ^2^ proposed fitting birth–death (BD) models to tuberculosis (TB) outbreak data using approximate Bayesian computation (ABC). Later on, the same setting was used in numerous ABC studies while the ABC methodology was being developed ^3– 8^. Stadler ^9^ and Aandahl *et al*. ^11^ also tested the ABC procedure against an exact Bayesian inference method based on an elaborate Markov Chain Monte Carlo (MCMC) sampling scheme. These investigations considered TB outbreak data from the San Francisco Bay area originally collected by Small *et al*. ^11^, who reported results from extensive epidemiological linking of the cases, as well as from the corresponding classical IS6110 fingerprinting genotypes. Such genetic data from the causative agent *Mycobacterium tuberculosis* are natural to characterize using the infinite alleles model (IAM), where each mutation is assumed to result in a novel allele in the bacterial strain colonizing the host. When lacking precise temporal information about the infection and the onset of the active disease, the numbers and sizes of genotype clusters can be used to infer the parameters of the BD model, as shown by Tanaka *et al*. ^2^ and Aandahl *et al*. ^10^.

Lintusaari *et al*. ^12^ demonstrated an issue with the nonidentifiability of *R* for the TB outbreak model in cases when both the birth and the death rates were unknown in the underlying birth–death process. This was visible as a nearly flat approximate likelihood over the parameter space of *R*. Additionally, they found that in cases when *R* was identifiable, the acquired estimate was dependent on the assumed population size *n*. In an earlier investigation by Tanaka *et al*. ^2^, a large infectious population size of *n* = 10,000 was required for the BD simulator to produce similar levels of genetic diversity to those observed in the San Francisco Bay data. Because it has not been observed, this assumption is difficult to justify when the acquired estimates depend on it.

Here we introduce an alternative formulation of the BD model that resolves the identifiability issue of *R*.

The proposed model does not require any assumptions about the underlying infectious population size, instead providing an estimate for that value as a byproduct of the inference. The model incorporates epidemiological knowledge about the TB infection and disease activation processes by assuming that the observed genotype data represent a mixture of three birth–death processes, each with clearly distinct characteristics. The new formulation depends on partially different parametrization, for which estimates can be found in the literature. By evaluating the ABC inference results of our model against the backdrop of the epidemiological information available in Small *et al*. ^11^, we see that both the significantly reduced infectious population size *n* and the increased *R* for the main driver component of the model make good sense. Our model thus provides a drastically different interpretation of these parameters than the ones offered by earlier studies.

In the new model, we consider latent and active TB infections separately, as only the latter lead to new transmission events. Transmission clusters are formed by recent infections that rapidly progress to active TB and spread further through the host population. Due to the rapid onset of symptoms in a new active case, the fingerprint of the pathogen remains the same throughout the transmission process, and its patients consequently form an epidemiological cluster. If, on the other hand, an infection remains latent, the pathogen undergoes mutations and thus acquires a new genetic fingerprint over the years ^11^. Through this and other epidemiologically motivated modelling choices, we show that the model becomes identifiable. Due to the rather modest requirements for the available data and the flexibility of modelling in ABC, our BD model could be applied to many similar settings beyond the case study considered in this article.

## 2. The model

Our model is based on a birth–death (BD) process in which a birth event corresponds to the appearance of a new case of active TB and a death event corresponds to any event that makes an existing host non-infectious. Such events include death, sufficient treatment, quarantine, and relocation away from the community under investigation. The model incorporates two BD processes and one pure birth process that have epidemiologically based interpretations. As in a standard BD process, these events are assumed to be independent of one another and to occur at specific rates. The time between two events is assumed to follow the exponential distribution specified by the rate of occurrence, causing the number of events to follow the Poisson distribution. The timescale considered here is one calendar year. The evolution of the infectious population is simulated by drawing events according to their rates.

Building upon the BD process, the simulated population carries auxiliary information. At birth, each case is assigned a cluster index that represents the specific genetic fingerprint of the pathogen and determines the cluster the case belongs to. The simulated output includes the cluster indexes that are recorded when cases are observed.

We will now explain our model in more detail and point out differences between it and the model of Tanaka *et al*. ^2^.

**Figure 1.**
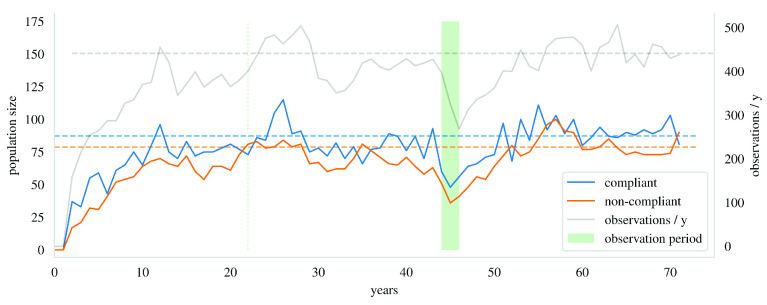
An illustration of simulated compliant and non-compliant populations as observed at the end of each year. Note that sampling can be done at any point once the model has stabilized; the drop in population sizes at the sampling point in this figure is purely coincidental.

First, we assume that observations are collected within a given time interval that matches that of the observed data. In the case of the San Francisco Bay data, the length of this interval is two years ^11^. Observations are collected from the simulated process after a sufficient warmup period so that the process can be expected to have reached stable properties. This procedure is visualized in Figure 1.

In the figure, the dashed lines are the balance values. The population sizes fluctuate around them after the process has matured. Both populations surpass their balance values at least once by the 22-year mark. The observation period is the green patch. The grey line shows the number of observations collected during each year of the simulation. The number of observations from the observation period and the clustering structure of the observations are used in the inference of the epidemiological parameters. A patient becomes observed in the study with probability *p _obs_*. Our model makes the simplifying assumption that both being observed and ceasing to be infectious are combined under the death event in the simulation. This is based on the assumption that a typical patient is treated promptly after being diagnosed ^13^, but we still allow for the possibility that some patients do not comply with treatment and remain infectious (see below). In contrast to the model of Tanaka *et al*. ^2^, there is no separate observation sampling phase, nor is there a prior estimate for the underlying population size.

We introduce a burden parameter *β* that reflects the rate at which new active TB cases with a previously unseen pathogen fingerprint appear in the community. This is the pure birth process of the model, and it represents reactivation of TB from latent cases as well as new pathogen fingerprints introduced by immigration. In the simulation, each such case receives a new cluster index that has not been assigned to any earlier case. Unlike Tanaka *et al*. ^2^, we do not explicitly model mutations. Instead, we assume they occur during the latent phase of infection over the years ^11^. This decision was partially motivated by the fact that Aandahl *et al*. ^10^ found the mutation rate parameter from 2 to be non-identifiable from the fingerprint data, and they consequently fixed that value to a constant.

We introduce two distinct birth–death processes for cases that are either *compliant* or *non-compliant* with treatment. These birth–death processes are parametrized with birth rates *τ _i_* and death rates *δ _i_*, where *i* = 1 denotes the non-compliant population and *i* = 2 the compliant population. A significant number of cases in the largest clusters observed by Small *et al*. ^11^ corresponded to non-compliant patients who stayed infectious for several months and belonged to subgroups under increased risk of rapid development of active TB due to conditions such as AIDS and substance abuse. Patients who are compliant with therapy typically cease being infectious quickly and do not transmit the disease as effectively as before diagnosis and treatment. Meta-analysis of typical time delays before diagnosis can be found in Sreeramareddy *et al*. ^13^.

We assume that a new TB case is non-compliant with therapy with probability *p*_1_. At transmission (birth event in the simulation), this probability is used to determine the patient type of the new case. We also assume that the epidemic is at a steady state ( Figure 1) by requiring that compliant cases have a reproductive number *R*_2_ = *τ*_2_ */δ*_2_ *<* 1 and that the reproductive number *R*_1_ of the non-compliant cases is constrained such that the population does not grow without limit. The steady state assumption is motivated by the tuberculosis incidence counts in the United States during the data collection period ^14^. In the next section, we identify the subspace of parameter values *R*_1_ and *R*_2_ that conform to this assumption.

## 3. Statistical analysis of the model

Let subscript *i* = 1 denote the non-compliant subpopulation and *i* = 2 the compliant subpopulation. We can analyze the sizes of these subpopulations by investigating the parameters of the three birth–death processes in the model. First, we notice that the size of a subpopulation follows a compound birth–death process whose birth rate is a linear function of the burden rate and of the birth rates of the two subpopulations at their respective present sizes. For instance, the birth rate of the non-compliant subpopulation is *p*_1_( *β* + *τ*_1_ *n*_1_ + *τ*_2_ *n*_2_), where *n*_1_ and *n*_2_ are the current subpopulation sizes and *p*_1_ is the probability of a case being non-compliant. The corresponding death rate is *δ*_1_ *n*_1_. Using this approach, we can determine the balance sizes *b*_1_ and *b*_2_ of the subpopulations—that is, the values of *n*_1_ and *n*_2_ that make the birth rate equal to the death rate in each subpopulation. In this steady state, the subpopulation sizes neither shrink nor grow. We obtain expressions for *b*_1_ and *b*_2_ by solving the following set of linear equations:

$$\begin{array}{l} \delta_{1}b_{1}=p_{1}(\beta +\tau_{1}b_{1}+\tau_{2}b_{2})\\ \delta_{2}b_{2}=p_{2}(\beta +\tau_{1}b_{1}+\tau_{2}b_{2}), \end{array}\;(1)$$

where *p*_2_ = 1 – *p*_1_ is the probability of a new case being compliant. The linear equations yield the following solution:

$$\begin{array}{l} b_{1}\;=\;\frac{p_{1}\;\beta \;\delta_{2}}{\delta_{2}\delta_{1}\;–p_{2}\tau_{2}\delta_{1}\;–\;p_{1}\tau_{1}\delta_{2}}\\ b_{2}\;=\;\frac{b_{1}(\delta_{1}\;–p_{1}\tau_{1})\;–\;p_{1}\beta }{p_{1}\;\tau_{2}}. \end{array}\;(2)$$

Given this solution, the balance values *b*_1_ and *b*_2_ exist when

$$\begin{array}{l} R_{1}\;<\;1/p_{1}\\ \;\mathrm{and} \end{array}\;(3)$$

$$\begin{array}{l} R_{2}\;<\;(1–p_{1}R{\;}_{1})/p_{2}\;. \end{array}\;(4)$$

Assuming, for instance, that *p*_2_ = 0.95 (as in 11), we would have *R*_1_ *<* 20.

Equations (2) also allow us to approximate the mean number of observed cases per year. We define this approximation as

$${\hat{n}}_{obs}\;=\;p_{obs}\;(\delta_{2}b_{2}\;+\;\delta_{1}b_{1}).\;(5)$$

Figure 1 illustrates how the population sizes fluctuate near their balance values in the simulation after a sufficient warmup period.

### 3.1 Parameter inference

We used approximate Bayesian computation to carry out parameter inference due to the unavailability of the likelihood function. This is the same approach used by Tanaka *et al*. ^2^ with the original model. The result is a sample from the approximate posterior distribution $\tilde{p}$ ( *R*_1_, *t*_1_, *R*_2_, *β | y*_0_) (see e.g. 18).

We used the Engine for Likelihood-Free Inference (ELFI) software ^15^ to perform our inference. Using rejection sampling, we selected 1000 parameter values from a total of 6M simulations. This large number of simulations was possible due to the fact that we implemented a computationally efficient, vectorized version of the simulator in Python. When we began this project, it was not possible to perform Bayesian optimization with non-uniform priors in ELFI, and so we utilized rejection sampling in order to incorporate priors appropriate to our model structure. A visualization of our ELFI model can be found in Figure S1 in this article’s Supplementary material. The observed data are available in 11. We have released the source code of our simulator and the corresponding ELFI model on GitHub ^1^. These resources allow for replication of our study.

***3.1.1 Priors.*** We set priors over the burden rate *β*, reproductive numbers *R*_1_ and *R*_2_, and the net transmission rate *t*_1_ = *τ*_1_ *− δ*_1_ of the non-compliant subpopulation. For the compliant population, we fix the death rate to an estimated *δ*_2_ = 5.95 (the total delay estimate; see 13). This value is used to calculate the net transmission rate *t*_2_ = *δ*_2_( *R*_2_ *−*1). Given the severity of the symptoms of active TB and bearing in mind the stringent protocols followed by public health officials, it is expected that virtually all active cases in the San Francisco Bay area were documented during the outbreak described in 11. Those data contained 585 confirmed cases of TB, of which 487 were included in that study. To account for the cases that were excluded, we fix the probability of being observed to *p _obs_* = 0.8. We set the probability of a new case being non-compliant to *p*_1_ = 0.05 (see p. 1708 of 11).

We give the burden rate *β* an informative prior that is able to produce a sufficient number of clusters with respect to the observed data. Specifically, we choose

β∼N(200,30).(6)

We give the net transmission rate *t*_1_ a uniform prior over a large interval from 0 to 30. Given the condition imposed by Inequality (3), we assign *R*_1_ and *R*_2_ uniform priors over a subspace that ensures the process has a steady state. More specifically,

$$\begin{array}{l} \;R_{1}\;∼\;\mathrm{Unif}(1.01,20),\\ R_{2}\;|R_{1}\;∼\;\mathrm{Unif}(0.01,(1–0.05\cdot R_{1})/0.95),\\ \;\mathrm{and}\\ \;t_{1}\;∼\;\mathrm{Unif}(0.01,30), \end{array}\;(7)$$

Given the observed data, we set the following additional constraints to optimize computation:

$$\begin{array}{l} {\hat{n}}_{obs}\;<\;350\\ \;\mathrm{and}\\ \;\tau_{1\;}\;<\;40. \end{array}\;(8)$$

**Figure 2.**
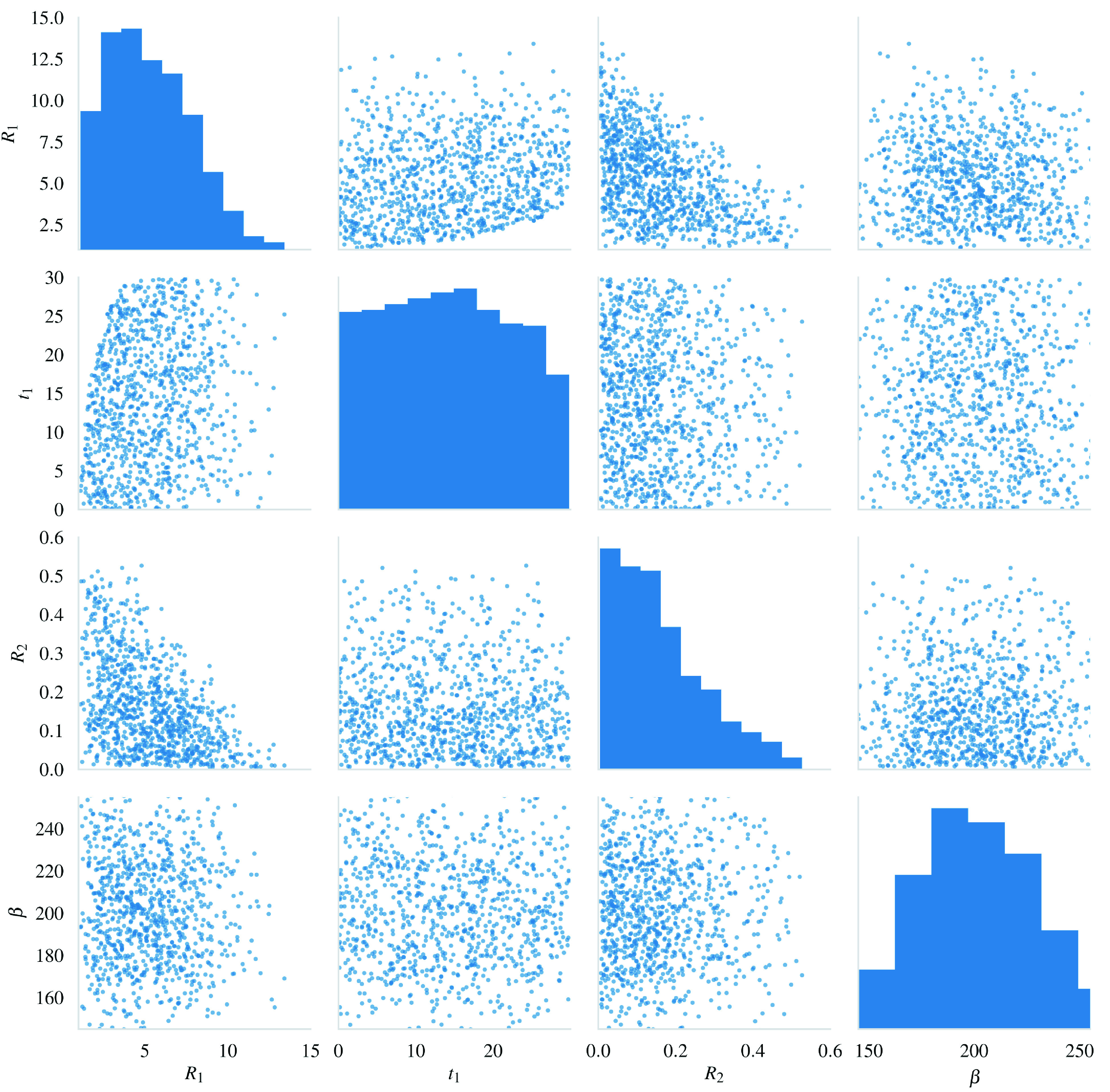
A scatter matrix of samples from the prior.

We verified that these constraints have a negligible effect on the acquired estimates. Their function is to prevent simulations with extremely unlikely parameter values, which saves a considerable amount of computation time. As a result of these constraints, all obtained estimates of *R*_1_ are smaller than 15. Figure 2 shows the samples drawn from the priors under these conditions.

***3.1.2 Summary statistics.*** The summary statistics used in earlier approaches (e.g. 2 and 12) are not directly applicable to our model. This is due to differences between the models that cause, for example, the number of observations in the sample to vary rather than being fixed. However, the previous studies’ summaries did prove to be a good starting point for the development of a more comprehensive set of summaries for our setting.

To that end, we ran extensive test simulations, performing inference on synthetic data generated by our model. From these simulations, we identified which summary statistics corresponded to appropriate behavior and ultimately selected eight of them. This decision was informed by observations about summary statistic behavior in 12, which presented a different model but utilized the same data that we use here.

These summary statistics aim to capture meaningful properties of the observed data given the new model. The first summary is the *number of observations*, which is here allowed to vary. Five of the summaries are related to the clustering structure, where a cluster is defined as a group of TB cases with the same genetic fingerprint: *the total* *number of clusters*, *the relative number of singleton clusters*, *the relative number of clusters of size two*, *the size of the largest cluster*, and *the mean of the successive differences in size among the four largest clusters* (see Table 1). These were chosen specifically to emphasize the most stable properties of the clustering structure. For instance, there is a substantial number of clusters of sizes one and two compared to those of other sizes. The relative number is used to remove the effect of variability in the numbers of observations and clusters between simulations.

**Table 1.** The summary statistics, their weights, and their values for the observed data y _0_.

Summary statistic	Explanation	Weight	*y*_0_
*n _obs_*	Number of observations.	1	473
*n _clusters_*	Number of clusters.	1	326
*r _c_*_1_	Relative number of singleton clusters. Computed as *r _c_*_1_ = *n _c_*_1_/ *n _obs_*, where *n _c_*_1_ is the number of clusters of size 1. The value of *r _c_*_2_ is computed likewise.	100/0.60	0.60
*r _c_*_2_	Relative number of clusters of size 2.	100/0.04	0.04
largest	Size of the largest cluster.	2	30
mean_largest_diff	Mean of the successive differences in size among the four largest clusters.	10	6.67
month_period	Number of months from the first observation to the last in the largest cluster.	10	24
obs_months	The number of months in which at least one observation was made from the largest cluster.	10	17

The remaining two summaries are related to the observation times of the largest cluster. Observation times were not included in earlier studies, and here they prove useful for identifying the net transmission rate *t*_1_. The summary statistics in question are *the number of months from the first observation to the last* and *the number of months in which at least one observation was made from the largest cluster*. It was possible to extract these data from figure 2 in 11. With these summaries, we aim to capture the span and rate at which transmissions occur.

It should be noted that the summaries chosen here do not consider global sufficiency (see e.g. 16). In cases where the dataset is very different from the San Francisco data, a modified set of summaries should probably be considered. Our distance function is the Euclidean distance between the weighted summary statistics of the observed and simulated data ( Table 1).

We weighted our summary statistics to adjust for and even out differences in their magnitudes. The final summary statistics and weights perform well in the evaluation of the model in subsection 4.1. The resulting acceptance/rejection threshold is *ε* = 31.7, while the smallest distance observed in our simulations is 12.5. Like our summary statistics, this threshold was selected from our trial runs of inference on synthetic data. We chose a value that struck a good balance between run time, acceptance rate, and the resulting Monte Carlo error rate.

## 4. Results

**Figure 3.**
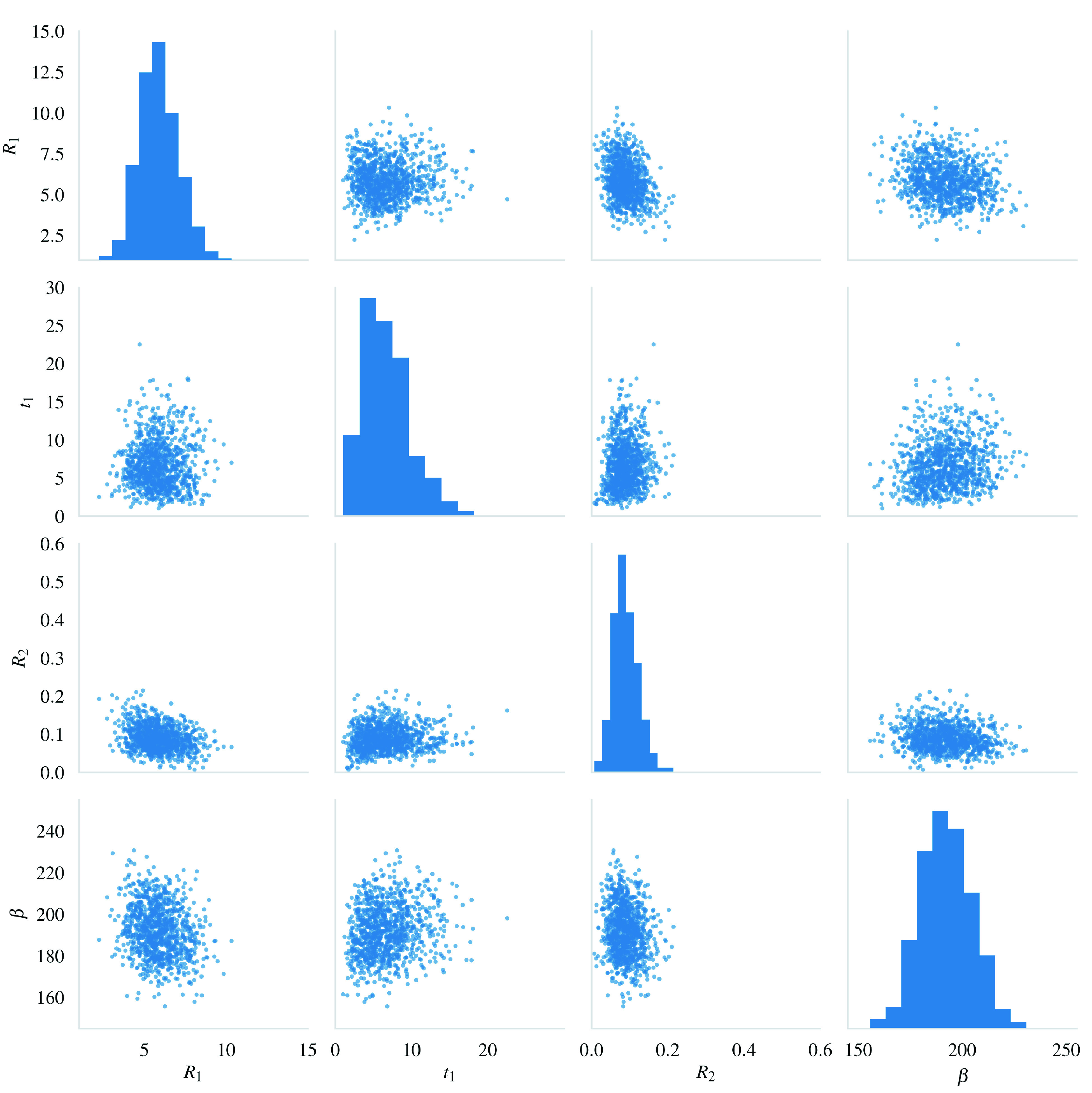
Posterior sample of size 1000 from the approximate posterior distribution $\tilde{p}$ ( *R*_1_, *t*_1_, *R*_2_, *β | y*_0_) plotted as a scatter matrix. Compare to the prior in Figure 2.

Figure 3 shows a sample of 1000 values from the joint approximate posterior distribution $\tilde{p}$ ( *R*_1_, *t*_1_, *R*_2_, *β | y*_0_). The pairwise sample clouds seem reasonably concentrated, do not extend to the edges of the axes, and are located inside the support of the prior ( Figure 2). The histograms and scatter plots are fairly normally shaped, with the only minor exception being that the net transmission rate of the non-compliant population *t*_1_ has a slight tail towards high values. A visual comparison of the posterior against the prior, together with the above observations, suggests that the model is identifiable for the San Francisco dataset.

The posterior means, medians and 95% credible intervals are given in Table 2. The means and medians are similar to one another, which indicates that the posterior distributions are symmetrical. *t*_1_ has the largest discrepancy due to the presence of the small tail mentioned above.

**Table 2.** Posterior summaries.

Parameter	Mean	Median	95% CI
*R*_1_	5.88	5.79	(3.68, 8.16)
*t*_1_	6.74	6.25	(1.57, 12.9)
*R*_2_	0.09	0.09	(0.03, 0.15)
*β*	192	192	(170, 216)

### 4.1 Evaluating the model identifiability

To further evaluate the reliability of the acquired estimates, we compute the mean and median absolute errors (MAE and MdAE) of the mean as well as the coverage property ^17^. These results include the ABC approximation error (see e.g. 18) caused by the summary statistics and the threshold of 31.7.

Table 3 lists the MAE and MdAE with the 95% error upper percentile for each parameter estimate. This information is useful for quantifying how much each estimate deviates from the actual parameter value on average. The burden rate ( *β*) and the reproductive number of the non-compliant population ( *R*_1_) have the smallest relative MAEs: 4.0% and 14.9%, respectively. The reproductive number of the compliant population ( *R*_2_) and the net transmission rate of the non-compliant population ( *t*_1_) have MAEs of 29.5% and 44.2%, respectively. The MAE of the latter seems rather high. The 95% percentile indicates that in 5% of the trials, the error was substantial. Further investigation of this issue shows that for some of the synthetic datasets, *t*_1_ is not identifiable, meaning that the synthetic data in those cases is not informative enough to produce a clear mode for the parameter. *R*_2_ suffered slightly from the same problem. This kind of situation, where some of the synthetic datasets turn out uninformative, is rather common when little data is available. Because of these exceptions, the MdAE might be a more appropriate measure than the MAE, as the former is not as heavily influenced by the results of non-identifiable datasets in trials. The relative MdAE errors for *R*_2_ and *t*_1_ were 21.9% and 32.1%, respectively.

**Table 3.** Mean and median absolute errors for 1000 trials with synthetic data from the posterior.

Parameter	MAE	Relative MAE ^2^	MdAE	Relative MdAE	95% percentile
*R*_1_	0.85	14.9%	0.72	12.6%	2.00
*t*_1_	2.68	44.2%	1.98	32.1%	7.66
*R*_2_	0.024	29.5%	0.018	21.9%	0.07
*β*	7.6	4.0 %	6.1	3.1%	19.8

**Figure 4.**
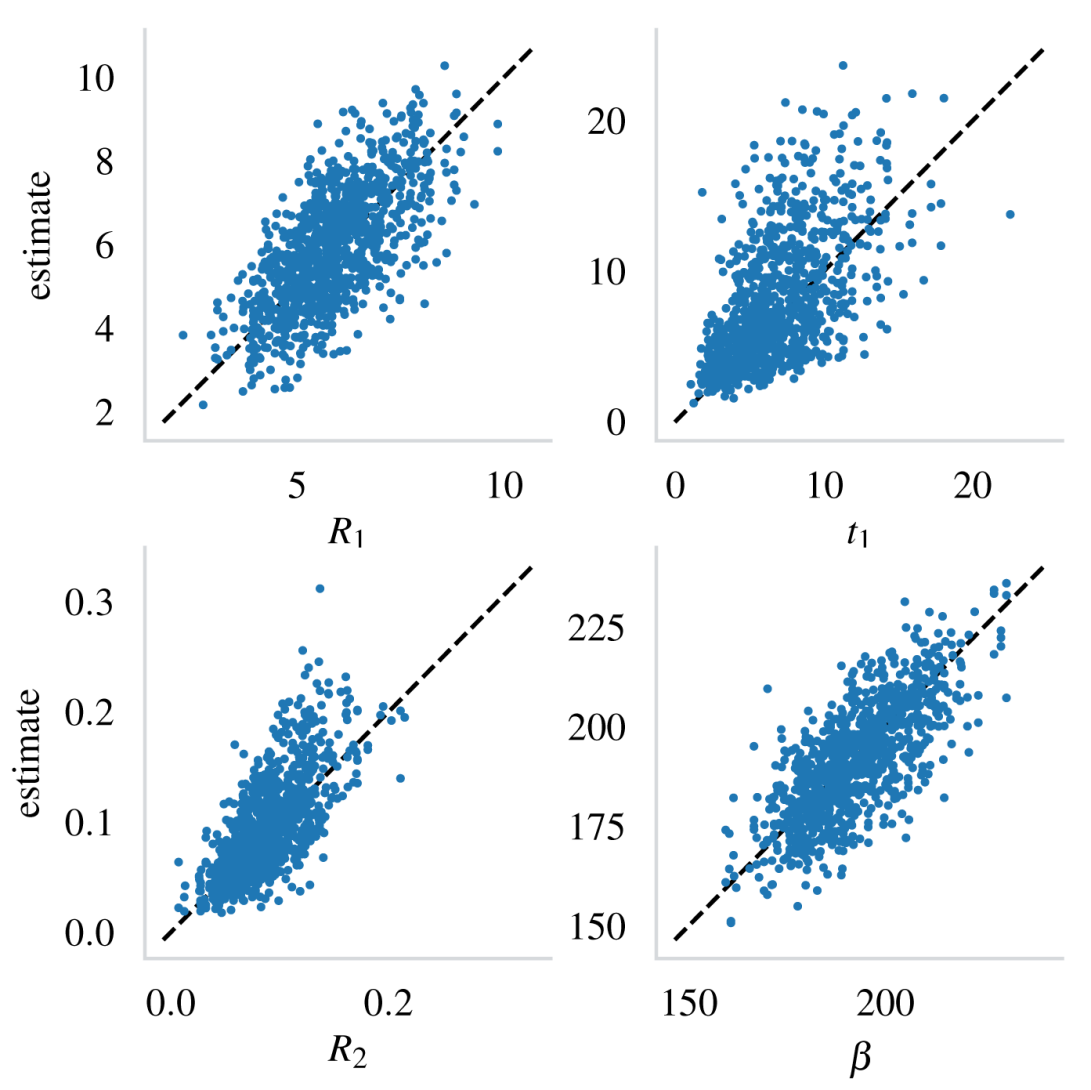
The estimates from the 1000 trials plotted against their true values. The black dashed line shows the 1:1 correspondence.

Figure 4 visualizes the estimated vs. actual values of each of the parameters.

Though *t*_1_ is only weakly identifiable, the results of our simulations indicate that the set of epidemiological parameters we have analyzed is identifiable for the San Francisco Bay dataset. Our simulations suggest that a structural model issue could be at fault for the weak identifiability of *t*_1_, as this can arise as a consequence of the generating stochastic process producing a relatively flat cluster distribution. Fortunately, *β* , *R*_1_ and *R*_2_, all of which provide more valuable epidemiological insight than *t*_1_, are robust against this identifiability issue.

The coverage property ^17^ is used to assess the reliability of the inference by checking whether the spreads of the acquired posterior distributions are accurate. Given a critical level *α*, the true parameter value should be outside the 1 *−α* credible interval of the posterior with probability *α*. We carried out our coverage analysis as follows.

First, we used rejection sampling to produce a sample for the posterior from the observed data. From this posterior, we sampled 1000 parameter vectors (with replacement) for the trials. For each of these 1000 vectors, we simulated synthetic data and performed rejection sampling in order to acquire posterior samples for that data. We then calculated the MAE for these 1000 trials. Finally, we applied the coverage property by determining in how many of the 1000 trials the original parameter was in the credible interval of the marginal posterior acquired from the synthetic data.

**Figure 5.**
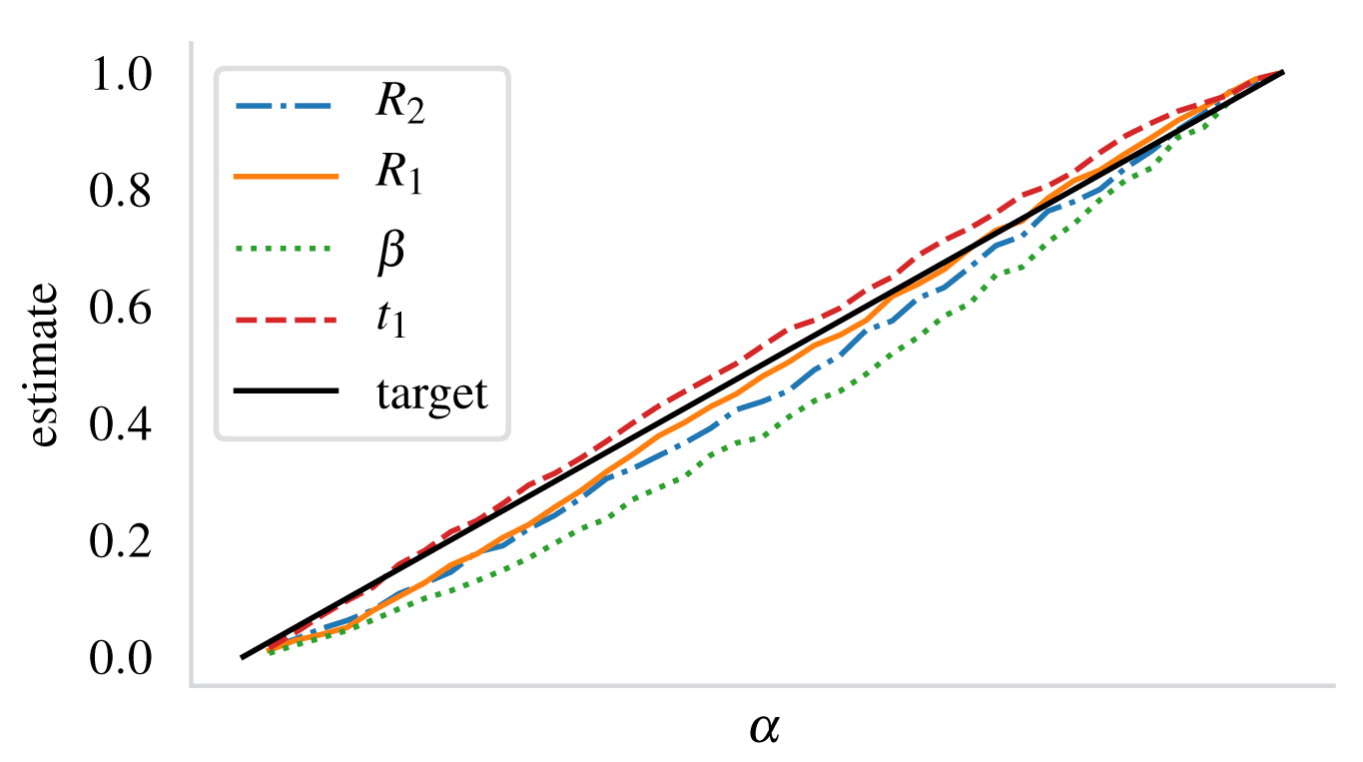
Mean estimates for the critical level *α* at different levels. The estimates are computed from 1000 synthetic datasets from the posterior. At *α* = 0.05, the estimates obtained for the parameters in the legend are, in order, 0.030, 0.028, 0.020 and 0.041.

The estimated *α* values from the 1000 marginal posteriors with known true parameter values appear satisfactory ( Figure 5). For the critical level *α* = 0.05, the estimated *α* values are ( *α _R_*_2_, *α _R_*_1_, *α _β_*, *α _t_*_1_) = (0.03, 0.03, 0.02, 0.04). The overall performance for different values of *α* was similar to this case in the sense that *α _β_* suffered from a larger error than the other parameters’ estimates ( Figure 5). Note that ABC coverage is not expected to be perfect due to the need for a credible interval.

## 5. Discussion

We have proposed a stochastic birth–death model to expand on several previous studies examining the use of simulator-based inference to investigate the spread of active TB within a community. Outbreaks of TB are characterized by epidemiologically linked clusters of patients with active TB that emerge within a relatively short time interval. The construction of our extended model was motivated by several observations made by Small *et al*. ^11^ concerning the San Francisco Bay transmission cluster data. There, the largest clusters tended to be founded by non-compliant patients. In the largest cluster, one such patient apparently infected 29 additional patients.

Earlier approaches ^2, 10^ suffered from the inability to reproduce these large clusters with an appropriate level of heterogeneity in cluster sizes without the prior assumption of a very large infectious population (to the order of 10,000 individuals) ^2, 12^. This assumption has a considerable effect on the estimate of the reproductive number *R*. However, epidemiological knowledge of TB does not support the existence of such a large infectious population in the study region during the observation period. Under our model, a prior estimate of the infectious population size is not needed. This model has a different parametrization for which estimates can be found from the literature. As a byproduct of the inference, the model also yields estimates for the infectious population size at the end of the data collection period. For the San Francisco Bay data, we found that the mean and median sizes of the compliant subpopulation were 48.4 and 48, respectively. The equivalent estimates for the non-compliant subpopulation were 13.5 and 11. These values are consistent with the findings of Small *et al*. ^11^.

For each subpopulation, the basic reproductive number ( *R*_1_ or *R*_2_) represents the average number of infections caused by a single infectious case that rapidly progresses to active TB. This value excludes latent infections, which are indirectly captured via the burden rate parameter *β*. We estimate that the basic reproductive number of non-compliant patients is *R*_1_ = 5.88 with a 95% credible interval (CI) of (3.68, 8.16); see Table 2. This estimate is nearly three times the one obtained by Aandahl *et al*. ^10^ with the same data, *R*_1_ = 2.10, which served as a blanket estimate for the whole infectious population (a distinction was not drawn between patient types). Our larger value would reasonably explain the formation of large clusters over a short time period. We estimate the reproductive number of the compliant subpopulation to be *R*_2_ = 0.09 with a 95% CI of (0.03, 0.15).

The ability of the proposed model to estimate *R*_1_ and *R*_2_ together with the infectious population size follows from several important changes we implement. One of them is to collect observations over a time span that matches the length of the actual observation period. In earlier work, observations were collected as a snapshot at a single point in time, which required that all patients in a large cluster be infectious simultaneously. However, in reality, observations are made over time as the outbreak evolves, and patients have different infectious periods. Figure 2 in 11 shows how patients were diagnosed at different times over their observation period. Another improvement in our model is the inclusion of a non-compliant patient type, which more closely reflects the findings of Small *et al*. ^11^ and enables the formation of heterogeneity in cluster sizes.

In our model, being compliant or non-compliant characterizes a patient’s type, and the model classifies each case at the time of the birth event. In reality, non-compliant patients are often diagnosed (i.e. observed) before they cease to be infectious, which implies that this simulator model deviates slightly from the real-world observation process. However, considering that this discrepancy applies to only roughly 5% of all observed cases, we do not expect it to cause significant bias. Furthermore, our summary statistics do not depend on exact diagnosis times: they rely instead on the span and the rate at which diagnoses occur.

Our model’s identifiability was found to be satisfactory for the San Francisco Bay dataset ( Figure 3). The relative mean absolute error in the estimate of *R*_1_ was 14.9% (0.85 in absolute terms; see Table 3). The same value for *R*_2_ was 29.5% (0.024 absolute). However, as discussed earlier, it is probably more sensible to use the median error (21.9%; 0.018 absolute) for *R*_2_. Coverage property analysis ^17^ suggests that the credible intervals produced by this model are reasonable. In future work, it would be interesting to evaluate the sensitivity of the model to other choices of literature-based parameter estimates.

As IS6110 typing remains in use despite advances in whole-genome sequencing of TB isolates, our model could be especially useful for investigations in middle- and low-income countries, where TB burden is often the highest. For example, the acquired estimates of epidemiological parameters could be used to gain insight into the relative efficacy of control programs across multiple communities. Given the apparent success of resolving the non-identifiability issue for *R* and removing the dubious assumption of an *a priori* known infectious population size by extending the BD model with relevant epidemiological knowledge from the literature, it would be interesting to generalize this approach to other pathogens for which the sampling process or other factors make simulator-based inference the most promising estimation method.

## Data availability

The observed data are available in 11.

Figure S1 is available at https://doi.org/10.6084/m9.figshare.7578728.v1.

## Software availability

**Source code:** https://github.com/jlintusaari/tb-model

**Archived source code at time of publication:** https://doi.org/10.5281/zenodo.2540933

**License:** 3-Clause BSD license

## Footnotes

^1^ https://github.com/lintusj1/tb-model
