# Supplementary material for: Resolving outbreak dynamics using approximate Bayesian computation for stochastic birth–death models
Source: Wellcome Open Res. 2019 Aug 30;4:14. Originally published 2019 Jan 25. [Version 2] doi: 10.12688/wellcomeopenres.15048.2 (PMC10514576; doi:10.12688/wellcomeopenres.15048.2)
Supplement: Peer review history

# Reviewer report: Reviewer response for version 2

**DOI:** 10.21956/wellcomeopenres.16856.r36361

**Published:** 2020-01-24

**Authors:** Mark Beaumont

**Affiliations:** 1 School of Biological Sciences, University of Bristol, Bristol, UK

The authors have very carefully addressed my comments, and I am happy with the current version.

Is the rationale for developing the new method (or application) clearly explained?

Partly

Is the description of the method technically sound?

Yes

Are the conclusions about the method and its performance adequately supported by the findings presented in the article?

Partly

If any results are presented, are all the source data underlying the results available to ensure full reproducibility?

Yes

Are sufficient details provided to allow replication of the method development and its use by others?

Yes

Reviewer Expertise:

Statistical population genetics

I confirm that I have read this submission and believe that I have an appropriate level of expertise to confirm that it is of an acceptable scientific standard.

# Reviewer report: Reviewer response for version 2

**DOI:** 10.21956/wellcomeopenres.16856.r36360

**Published:** 2019-10-07

**Authors:** Olivier Gascuel

**Affiliations:** 1 Unité Bioinformatique Evolutive, C3BI USR 3756 Institut Pasteur & CNRS, Paris, France

The authors have made changes that further improved the clarity and the flow of the manuscript and addressed our comments and suggestions. We propose final approval of the paper. We would like to suggest several minor changes not requiring any further analysis.

1. While adding a workflow of the study helps greatly, we think that adding a flow diagram of the model ( *i.e.* the flow of individuals between different states while pointing out individual rates, e.g. see Figure 1 at https://institutefordiseasemodeling.github.io/Documentation/general/model-seir.html for SEIR model) would help the readers to grasp quickly the mathematical model.
2. We believe that adding some parts of the original, more extensive, description of the Figure 1 explaining the meaning of dashed lines (balance values) should be kept.
3. “It should be noted that the summaries chosen here do not consider global sufficiency. In cases where the dataset is very different from the San Francisco data, a modified set of summaries should probably be considered.”
  With respect to this remark, we believe an extensive list of the summary statistics that you tried before identifying the final set, would help further research and adaptation of your method to similar studies. Such a list might be added as Supplementary Material.

Hope this helps, sincerely,

 Jakub Voznica, Anna Zhukova and Olivier Gascuel

Is the rationale for developing the new method (or application) clearly explained?

Yes

Is the description of the method technically sound?

Yes

Are the conclusions about the method and its performance adequately supported by the findings presented in the article?

Yes

If any results are presented, are all the source data underlying the results available to ensure full reproducibility?

Yes

Are sufficient details provided to allow replication of the method development and its use by others?

Yes

Reviewer Expertise:

NA

I confirm that I have read this submission and believe that I have an appropriate level of expertise to confirm that it is of an acceptable scientific standard.

# Reviewer report: Reviewer response for version 1

**DOI:** 10.21956/wellcomeopenres.16417.r34750

**Published:** 2019-04-12

**Authors:** Mark Beaumont

**Affiliations:** 1 School of Biological Sciences, University of Bristol, Bristol, UK

This interesting paper covers an area - TB epidemiology - that has been the subject of a number of papers that have used approximate Bayesian computation (ABC). These studies have also involved some full-likelihood MCMC solutions for the same models. The present paper carries out an ABC analysis with an alternative modelling framework, which provides some satisfactory solutions to some problems that had been previously noted.

 I generally have few quibbles with the ABC analysis. My only main query with the paper, in my ignorance of TB epidemiology, is to what extent the new modelling framework gives a reasonable representation of the underlying biology. I note that the original Tanaka *et al.* paper explicitly emphasised the importance of modelling the mutational structure of clusters (3rd paragraph of the introduction). By contrast the present paper gives no justification for dropping the mutational modelling. My reason for querying this is that, as I understand it, Tanaka had a previous history of detailed work on TB epidemiology, prior to the 2006 paper, including co-authorship with Peter Small, and so presumably was able to put in the benefit of that experience into the paper. Therefore I recommend that this aspect be much better justified and discussed.

 On the ABC side, my main query is to what extent the authors are confident about identifiability of their parameters. Particularly, since they seem to suggest one of their parameters is not identifiable (discussed more in specific points below). In a model-free setting, identifiability is demonstrated through simulation, rather than analytically. Obviously, if non-identifiability is shown this naturally leads to some questions about the summary statistics etc. as well as the structure of the model itself. But, with informative priors, some parameters that are only jointly identifiable can appear to be identifiable marginally - in a population genetics context the apparent identifiability of N and \mu with informative priors is a case in point, when only their product is identifiable. Again, this needs a bit more discussion than in the present paper.

Specific Comments:

- Introduction, first paragraph: "genotype fingerprints". Some more discussion of this would be useful with regard to my point above. Presumably what concerned Tanaka *et al.* is that multiple outbreaks can involve the same cluster, and that different clusters (due to mutation?) could arise from the same outbreak.
- Model, 4th paragraph: “p_{obs}” - does the assumption of being observed lead to ceasing to be infectious fit with the compliant/non-compliant distinction, two paragraphs further down?
- Model, 5th paragraph: Note my main query.
- Summary statistics, paragraph 2: It might be helpful to emphasise that a 'cluster' here is assumed to be a new active TB case. Presumably many of these summary statistics are highly correlated with the parameter \beta?
- Figure 2/3: I wonder whether these might be better in the supp. text, and replaced with a single figure with HPD contours for the prior and posterior.
- Summary statistics, last paragraph: "It is good to note". Do the authors mean that? Or rather do they mean "It should be noted"? Presumably it is not good to be not sufficient. More generally, is there an argument to use projections as in Fearnhead and Prangle (2012 ^1^), which are generally straightforward to apply? I think there are good reasons (Fearnhead and Prangle, 2012 ^1^; Li and Fearnhead, 2018 ^2^) for expecting the optimal number of summary statistics to be the same as the number of parameters, thus reducing the effect of the 'curse of dimensionality'.
- Results, 3rd paragraph: coverage property. The analysis seems fine, but the authors skirt some details, worth noting. They use 'true' values from the ABC posterior. The Wegmann *et al.* paper, following Cook *et al.*, simulated 'true' values from the prior, for which coverage is indeed uniform. It is not so obvious that coverage from the ABC posterior should also be uniform, but this is demonstrated (I think for the first time) in Prangle *et al.* (2014 ^3^) (at least for any interval in the prior predictive distribution of summary statistics, including the interval from which the ABC posterior is computed).
- Results, 4th paragraph: non-identifiability of t_1. This observation seems at variance with what is stated in the abstract. Is this a summary statistic issue? Or a structural model issue?
- Figure 5: These results look convincing. Note that because of the need for a tolerance interval ABC coverage is not expected to be perfect (Fearnhead and Prangle, 2012 ^1^).

Is the rationale for developing the new method (or application) clearly explained?

Partly

Is the description of the method technically sound?

Yes

Are the conclusions about the method and its performance adequately supported by the findings presented in the article?

Partly

If any results are presented, are all the source data underlying the results available to ensure full reproducibility?

Yes

Are sufficient details provided to allow replication of the method development and its use by others?

Yes

Reviewer Expertise:

Statistical population genetics

I confirm that I have read this submission and believe that I have an appropriate level of expertise to confirm that it is of an acceptable scientific standard, however I have significant reservations, as outlined above.

## References

[1] : Constructing summary statistics for approximate Bayesian computation: semi-automatic approximate Bayesian computation. *Journal of the Royal Statistical Society: Series B (Statistical Methodology)* .2012;74(3) : 10.1111/j.1467-9868.2011.01010.x 419-474 10.1111/j.1467-9868.2011.01010.x

[2] : On the asymptotic efficiency of approximate Bayesian computation estimators. *Biometrika* .2018;105(2) : 10.1093/biomet/asx078 285-299 10.1093/biomet/asx078

[3] : Diagnostic tools for approximate Bayesian computation using the coverage property. *Australian & New Zealand Journal of Statistics* .2014;56(4) : 10.1111/anzs.12087 309-329 10.1111/anzs.12087

# Response

**Published:** 2019-07-30

**Authors:** Jarno Lintusaari

**Affiliations:** Aalto University School of Science and Technology, Finland

*We thank the reviewers for their useful comments that allowed us to improve the paper. Below we provide detailed responses to the issues brought up. Our responses are written in italics.*

This interesting paper covers an area - TB epidemiology - that has been the subject of a number of papers that have used approximate Bayesian computation (ABC). These studies have also involved some full-likelihood MCMC solutions for the same models. The present paper carries out an ABC analysis with an alternative modelling framework, which provides some satisfactory solutions to some problems that had been previously noted.

I generally have few quibbles with the ABC analysis. My only main query with the paper, in my ignorance of TB epidemiology, is to what extent the new modelling framework gives a reasonable representation of the underlying biology. I note that the original Tanaka et al.paper explicitly emphasised the importance of modelling the mutational structure of clusters (3rd paragraph of the introduction). By contrast the present paper gives no justification for dropping the mutational modelling. My reason for querying this is that, as I understand it, Tanaka had a previous history of detailed work on TB epidemiology, prior to the 2006 paper, including co-authorship with Peter Small, and so presumably was able to put in the benefit of that experience into the paper. Therefore I recommend that this aspect be much better justified and discussed.

*This is a very relevant point. However, the subsequent joint work by the Tanaka et al. authors and Tanya Stadler notified that the mutation parameter appears non-identifiable from the fingerprint data and used a fixed value obtained from the literature in their most recent paper. We have now included a discussion about this and the rationale for excluding an explicit mutation rate parameter in our model.*

On the ABC side, my main query is to what extent the authors are confident about identifiability of their parameters. Particularly, since they seem to suggest one of their parameters is not identifiable (discussed more in specific points below). In a model-free setting, identifiability is demonstrated through simulation, rather than analytically. Obviously, if non-identifiability is shown this naturally leads to some questions about the summary statistics etc. as well as the structure of the model itself. But, with informative priors, some parameters that are only jointly identifiable can appear to be identifiable marginally - in a population genetics context the apparent identifiability of N and \mu with informative priors is a case in point, when only their product is identifiable. Again, this needs a bit more discussion than in the present paper.

*We agree that an additional discussion is in place and have added such in the revision. Our simulation experiments reported in the paper suggest that the key epidemiological parameters are indeed identifiable for the SF Bay data set, even if the net transmission rate may remain only weakly identifiable.*

Specific Comments:

    Introduction, first paragraph: "genotype fingerprints". Some more discussion of this would be useful with regard to my point above. Presumably what concerned Tanaka et al. is that multiple outbreaks can involve the same cluster, and that different clusters (due to mutation?) could arise from the same outbreak.

*We have added further discussion. Noting the slow mutation rate of TB, it is highly unlikely that multiple clusters would arise from the same outbreak within a relatively short timespan.*

    

    Model, 4th paragraph: “p_{obs}” - does the assumption of being observed lead to ceasing to be infectious fit with the compliant/non-compliant distinction, two paragraphs further down?

*Good point, there was a sloppy phrasing in the 4 ^th^ paragraph. We have now revised the text to be in line with the later paragraph.*

     

    Model, 5th paragraph: Note my main query.

*As noted in our response to the main query item, we have now edited the text accordingly.*

    

    Summary statistics, paragraph 2: It might be helpful to emphasise that a 'cluster' here is assumed to be a new active TB case. Presumably many of these summary statistics are highly correlated with the parameter \beta?

*Excellent remarks, we have added further clarification about this.*

    

    Figure 2/3: I wonder whether these might be better in the supp. text, and replaced with a single figure with HPD contours for the prior and posterior.

*We do appreciate this suggestion, however, as noted in the response to R1*, *the first author who had the main responsibility for all aspects of the presented work has already graduated and left academia, as has the second author, so neither of the two are able to contribute to further work related to this paper. We would thus prefer keeping the two figures as in their current versions.*

    

    Summary statistics, last paragraph: "It is good to note". Do the authors mean that? Or rather do they mean "It should be noted"? Presumably it is not good to be not sufficient.

*The reviewer has a correct interpretation, this was a typo and is now fixed.*

More generally, is there an argument to use projections as in Fearnhead and Prangle (2012 ^1^), which are generally straightforward to apply? I think there are good reasons (Fearnhead and Prangle, 2012 ^1^; Li and Fearnhead, 2018 ^2^) for expecting the optimal number of summary statistics to be the same as the number of parameters, thus reducing the effect of the 'curse of dimensionality'.

*It is indeed correct that the number of summary statistics is generally expected to match the dimensionality of the parameter space. As noted in the response to R1,* *the summary statistics were iteratively defined by trialing inference on synthetic data from the model. The final set of statistics was settled on after extensive test simulations showing appropriate behavior. Note also that we had previous experience about the behavior of various summary statistics from the earlier Lintusaari et al. Genetics 2016 article examining inference for a different model but the same data.*

    

    Results, 3rd paragraph: coverage property. The analysis seems fine, but the authors skirt some details, worth noting.

*Detailed description of the simulations used to study the coverage property has been added.*

They use 'true' values from the ABC posterior. The Wegmann et al. paper, following Cook et al., simulated 'true' values from the prior, for which coverage is indeed uniform. It is not so obvious that coverage from the ABC posterior should also be uniform, but this is demonstrated (I think for the first time) in Prangle et al. (2014 ^3^) (at least for any interval in the prior predictive distribution of summary statistics, including the interval from which the ABC posterior is computed).

*Good point, we have added reference to Prangle et al. in the relevant part of the text.*

    

    Results, 4th paragraph: non-identifiability of t_1. This observation seems at variance with what is stated in the abstract. Is this a summary statistic issue? Or a structural model issue?

*We have edited the text to clarify the potential weak identifiability of t_1. Our simulations suggest that it is a structural model issue such that the parameter sometimes becomes only weakly identifiable when the generating stochastic process happens to result in a particularly flat distribution of clusters. Encouragingly, the other parameters, which are the most relevant ones from the epidemiological perspective, do appear fairly robustly identifiable even if t_1 would have a flat posterior.*

    

    Figure 5: These results look convincing. Note that because of the need for a tolerance interval ABC coverage is not expected to be perfect (Fearnhead and Prangle, 2012 ^1^).

*Good point, we have added a remark about this to the revised text.*

# Reviewer report: Reviewer response for version 1

**DOI:** 10.21956/wellcomeopenres.16417.r34679

**Published:** 2019-02-13

**Authors:** Olivier Gascuel, Jakub Voznica, Anna Zhukova

**Affiliations:** 1 Unité Bioinformatique Evolutive, C3BI USR 3756 Institut Pasteur & CNRS, Paris, France

**Article summary**

 The article describes a new model of TB outbreak in San Francisco Bay area that overcomes the non-identifiability/dependency on the assumed population size of the reproductive number R in the generic birth-death-mutation model by Tanaka *et al.  *The new model considers two compartments, for compliant and non-compliant subpopulations, and combines two birth-death processes (for each of the compartments) with a pure-birth process that creates new TB transmission clusters (i.e. a new individual with a new RFLP pattern that is further transmitted). This pure-birth process replaces mutation in Tanaka’s model and corresponds to migration or reactivating of a latent TB. The rate corresponding to the pure-birth process is referred as the burden rate. At each (non-burden) birth event (i.e. TB transmission) the compartment of the newly infected individual is assigned to non-compliant or compliant with the probability p1 or (1 - p1) correspondingly. At each death (i.e. becoming non-infectious) event the individual is sampled with the probability p-obs.

 Overall, the proposed model has 7 parameters: the burden rate, 2 birth rates, 2 death rates, and 2 probabilities (p1 and p-obs). However, 3 of them (compliant death rate, p-obs and p1) were fixed based on the estimates from the literature, therefore leaving 4 parameters to be estimated, expressed in terms of two reproductive numbers, i.e. birth to death rate ratios for the corresponding compartments, the non-compliant net transmission rate (difference between the birth and the death rates), and the burden rate. Priors and additional constraints on the rates were set to avoid biological meaningless of the simulations.

 The simulator was implemented for the proposed model and parameter estimation was performed for the data collected in SF Bay area in 1991-92 (Small et al.) with ABC, based on 1000 parameter values sampled with rejection from 6M simulations, using 8 (weighted) summary statistics:  

 1.    the number of observations

 2.    the total number of clusters

 3.    the relative number of singleton clusters

 4.    the relative number of clusters of size two

 5.    the size of the largest cluster

 6.    the mean of the successive difference in size among the four largest clusters

 7.    the number of months from the first observation to the last

 8.    the number of months when at least one observation was made.

**

 The new model not only allowed for estimation of the aforementioned parameters (posteriors are well concentrated within but far from the edges of the priors) but also of the balance subpopulation sizes (at the equilibrium state when infected subpopulations neither shrink nor grow). The estimates differ from those done with the birth-death-mutation model, and are potentially better aligned with the epidemiological knowledge on TB in the area.

 The coverage property (accuracy of the spread of the acquired posterior) of the estimator was further tested on 1000 parameter values drawn from the posterior, giving satisfactory results for the critical level of .05 (the true parameter values were outside of the .95 credible interval of the posterior with probability less than .05).

**General comments**

 The article reads well, the model, rationale behind it, its assumptions and advantages over the previous TB model are explained in a clear and convincing way. It is a valuable addition to TB research, and we believe that the article should be accepted.

 Having little knowledge on TB (but on ABC), we feel like the article could benefit from a more detailed discussion of the obtained estimates. For example, is there any literature/other data supporting the estimated subpopulation sizes?

 We also point out a few technicalities that could be explained in more detail (see below).

**Technical comments**

 A flow diagram of the model could facilitate the model understanding for the reader.

 Additional sensitivity analysis of the model while varying pre-fixed parameter values (of compliant death rate, p-obs and p1) might add confidence in author’s findings.

 Page 4: *“The observations are collected from the simulated process after a sufficient warm-up period, so that the process can be expected to have reached stable properties (exemplified in Figure 1).”*

 In Figure 1 the warm-up seems to be achieved already after 15 years, however the observation period is chosen around 45 years, where there is a drop of population sizes. Is it a coincidence? How is the start of the observation period selected?

 Page 5: *“We used the Engine for Likelihood-Free Inference (ELFI)...”*

 The authors might detail what kind of inference was used: Is it a pure distance/rejection-based approach? Or do you use some regression tool, random forest, LASSO, neural network or other? How was the technique selected?

 Page 5: *“Based on the details in Small et al. describing the San Francisco Bay area TB data, there were 585 confirmed cases of TB of which 487 were included in the study. To account for the cases that were not included in the study, we fix the probability of becoming observed to p-obs = 0.8”*

 If we understand correctly the p-obs is calculated as 487/585, but what about potentially unknown cases of TB in the SF Bay area? Is it assumed that all the existing TB cases are known?

 Page 5: It is not very clear why these particular summary statistics were selected, e.g. *“the mean of the successive difference in size among the four largest clusters”*

 Why not 3 or 5, etc.? Were for example other statistics tested, which performed worse?

 The name of the last statistic ( *“the number of months when at least one observation was made”*) is rather confusing. In table 1 it has a slightly different name: *“the number of months that at least one observation was made from the largest cluster”*. Does it mean *the time when the first observation from the largest cluster was made*?

 Page 7: *“The chosen summary statistics and weights were found to perform well in the evaluation of the model in Subsection .”*

 The subsection number is missing.

 Page 7: *“The resulting threshold for the acquired sample was* *∈* *= 31.7 with the smallest distance being 12.5.”*

 How were the threshold and distance values selected?

Is the rationale for developing the new method (or application) clearly explained?

Yes

Is the description of the method technically sound?

Yes

Are the conclusions about the method and its performance adequately supported by the findings presented in the article?

Yes

If any results are presented, are all the source data underlying the results available to ensure full reproducibility?

Yes

Are sufficient details provided to allow replication of the method development and its use by others?

Yes

Reviewer Expertise:

NA

We confirm that we have read this submission and believe that we have an appropriate level of expertise to confirm that it is of an acceptable scientific standard, however we have significant reservations, as outlined above.

# Response

**Published:** 2019-07-30

**Authors:** Jarno Lintusaari

**Affiliations:** Aalto University School of Science and Technology, Finland

*We thank the reviewers for their useful comments that allowed us to improve the paper. Below we provide detailed responses to the issues brought up. Our responses are written in italics.*

R1:

The article describes a new model of TB outbreak in San Francisco Bay area that overcomes the non-identifiability/dependency on the assumed population size of the reproductive number R in the generic birth-death-mutation model by Tanaka et al.  The new model considers two compartments, for compliant and non-compliant subpopulations, and combines two birth-death processes (for each of the compartments) with a pure-birth process that creates new TB transmission clusters (i.e. a new individual with a new RFLP pattern that is further transmitted). This pure-birth process replaces mutation in Tanaka’s model and corresponds to migration or reactivating of a latent TB. The rate corresponding to the pure-birth process is referred as the burden rate. At each (non-burden) birth event (i.e. TB transmission) the compartment of the newly infected individual is assigned to non-compliant or compliant with the probability p1 or (1 - p1) correspondingly. At each death (i.e. becoming non-infectious) event the individual is sampled with the probability p-obs.

Overall, the proposed model has 7 parameters: the burden rate, 2 birth rates, 2 death rates, and 2 probabilities (p1 and p-obs). However, 3 of them (compliant death rate, p-obs and p1) were fixed based on the estimates from the literature, therefore leaving 4 parameters to be estimated, expressed in terms of two reproductive numbers, i.e. birth to death rate ratios for the corresponding compartments, the non-compliant net transmission rate (difference between the birth and the death rates), and the burden rate. Priors and additional constraints on the rates were set to avoid biological meaningless of the simulations.

The simulator was implemented for the proposed model and parameter estimation was performed for the data collected in SF Bay area in 1991-92 (Small et al.) with ABC, based on 1000 parameter values sampled with rejection from 6M simulations, using 8 (weighted) summary statistics: 

1.    the number of observations

2.    the total number of clusters

3.    the relative number of singleton clusters

4.    the relative number of clusters of size two

5.    the size of the largest cluster

6.    the mean of the successive difference in size among the four largest clusters

7.    the number of months from the first observation to the last

8.    the number of months when at least one observation was made.

The new model not only allowed for estimation of the aforementioned parameters (posteriors are well concentrated within but far from the edges of the priors) but also of the balance subpopulation sizes (at the equilibrium state when infected subpopulations neither shrink nor grow). The estimates differ from those done with the birth-death-mutation model, and are potentially better aligned with the epidemiological knowledge on TB in the area.

The coverage property (accuracy of the spread of the acquired posterior) of the estimator was further tested on 1000 parameter values drawn from the posterior, giving satisfactory results for the critical level of .05 (the true parameter values were outside of the .95 credible interval of the posterior with probability less than .05).

**General comments**

The article reads well, the model, rationale behind it, its assumptions and advantages over the previous TB model are explained in a clear and convincing way. It is a valuable addition to TB research, and we believe that the article should be accepted.

*We thank the reviewers for their highly positive comments about our work.*

Having little knowledge on TB (but on ABC), we feel like the article could benefit from a more detailed discussion of the obtained estimates. For example, is there any literature/other data supporting the estimated subpopulation sizes?

*The estimates are well aligned with the epidemiological discussion in the original NEJM paper introducing the fingerprint data. We now point this out more carefully in the revised version.*

We also point out a few technicalities that could be explained in more detail (see below).

**Technical comments**

A flow diagram of the model could facilitate the model understanding for the reader.

*A flow diagram has been added as a supplementary figure to accompany the final version.*

Additional sensitivity analysis of the model while varying pre-fixed parameter values (of compliant death rate, p-obs and p1) might add confidence in author’s findings.

*We feel that the current sensitivity analysis is quite sufficient and fulfils its purpose to demonstrate stability of the estimates for data akin the San Francisco Bay observations. The first author who had the main responsibility for all aspects of the presented work has already graduated and left academia, so he is not able to contribute to further work related to this paper which limits our possibilities for performing extensive additional simulations.*

Page 4: “The observations are collected from the simulated process after a sufficient warm-up period, so that the process can be expected to have reached stable properties (exemplified in Figure 1).”

In Figure 1 the warm-up seems to be achieved already after 15 years, however the observation period is chosen around 45 years, where there is a drop of population sizes. Is it a coincidence? How is the start of the observation period selected?

*Figure 1 is intended only as a schematic numerical example to assist the reader in understanding the underlying logic of the model and the sampling assumptions. The drop is thus coincidental. This is now properly noted in the revision.*

Page 5: “We used the Engine for Likelihood-Free Inference (ELFI)...”

The authors might detail what kind of inference was used: Is it a pure distance/rejection-based approach? Or do you use some regression tool, random forest, LASSO, neural network or other? How was the technique selected?

*As stated in the paper, we used pure rejection sampling (we sampled 1000 parameter values with rejection sampling from a total of 6M simulations). The main reasons for choosing this basic ABC approach were: 1) we implemented a computationally efficient vectorized Python version of the simulator which facilitated the use of a large number of simulations, 2) at the time the project was initiated, ELFI had yet no implementation of the Bayesian optimization procedure for non-uniform priors. Such a prior was essential for the model structure and straightforward to consider in a pure ABC rejection sampler, hence the choice for inference method was well motivated. These reasons are now more clearly stated in the revision.*

Page 5: “Based on the details in Small et al. describing the San Francisco Bay area TB data, there were 585 confirmed cases of TB of which 487 were included in the study. To account for the cases that were not included in the study, we fix the probability of becoming observed to p-obs = 0.8”

If we understand correctly the p-obs is calculated as 487/585, but what about potentially unknown cases of TB in the SF Bay area? Is it assumed that all the existing TB cases are known?

*For epidemiological reasons it is unlikely that any substantial numbers of active TB cases were unknown to the public health officials, hence it is unlikely that these would have a non-negligible contribution to the observed outbreaks. Given the severity of TB and the protocols followed by public health officials most active cases are expected to have been traced. We have now stated this more explicitly in the revision.*

Page 5: It is not very clear why these particular summary statistics were selected, e.g. “the mean of the successive difference in size among the four largest clusters”

Why not 3 or 5, etc.? Were for example other statistics tested, which performed worse?

The name of the last statistic (“the number of months when at least one observation was made”) is rather confusing. In table 1 it has a slightly different name: “the number of months that at least one observation was made from the largest cluster”. Does it mean the time when the first observation from the largest cluster was made?

*We have edited the text to make the summary statistic definitions unambiguous. The summary statistics were iteratively defined by trialing inference on synthetic data from the model. The final set of statistics was settled on after extensive test simulations showing appropriate behavior. Note also that we had previous experience about the behavior of various summary statistics from the earlier Lintusaari et al. Genetics 2016 article examining inference for a different model but the same data.*

Page 7: “The chosen summary statistics and weights were found to perform well in the evaluation of the model in Subsection .”

The subsection number is missing.

*The subsection number was missing due to the submission template and will be visible in the final typeset version.*

Page 7: “The resulting threshold for the acquired sample was ∈ = 31.7 with the smallest distance being 12.5.”

How were the threshold and distance values selected?

*As for the summary statistics, the threshold was settled by extensive trialing of inference on synthetic data from the model to identify a threshold striking a good balance between runtimes and acceptance rate and the resulting Monte Carlo error rate. This is now more appropriately reported in the revision.*
